# Sempervirine inhibits RNA polymerase I transcription independently from p53 in tumor cells

**DOI:** 10.1038/s41420-020-00345-4

**Published:** 2020-10-28

**Authors:** Cinzia Caggiano, Eugenia Guida, Federica Todaro, Pamela Bielli, Mattia Mori, Francesca Ghirga, Deborah Quaglio, Bruno Botta, Fabiola Moretti, Paola Grimaldi, Pellegrino Rossi, Emmanuele A. Jannini, Marco Barchi, Susanna Dolci

**Affiliations:** 1 Department of Biomedicine and Prevention, University of Rome Tor Vergata, Rome, Italy; 2 Department of Biotechnology, Chemistry and Pharmacy, University of Siena, Siena, Italy; 3 Center for Life Nano Science@Sapienza, Istituto Italiano di Tecnologia, Rome, Italy; 4 Department of Chemistry and Drug Technology, University of Rome La Sapienza, Rome, Italy; 5 Institute of Cell Biology and Neurobiology, National Research Council of Italy (CNR), Rome, Italy; 6 Department of Systems Medicine, University of Rome Tor Vergata, Rome, Italy

**Keywords:** Testicular cancer, Target identification

## Abstract

In the search of small molecules that can target MDM2/p53 pathway in testicular germ cell tumors (TGCTs), we identified sempervirine (2,3,4,13-tetrahydro-1*H*-benz[*g*]indolo[2,3-*a*]quinolizin-6-ium), an alkaloid of *Gelsemium sempervirens*, that has been previously proposed as an inhibitor of MDM2 that targets *p53-wildtype* (*wt*) tumor cells. We found that sempervirine not only affects cell growth of *p53-wt* cancer cells, but it is also active in *p53-mutated* and *p53-null* cells by triggering p53-dependent and independent pathways without affecting non-transformed cells. To understand which mechanism/s could be activated both in *p53-wt* and *-null* cells, we found that sempervirine induced nucleolar remodeling and nucleolar stress by reducing protein stability of RPA194, the catalytic subunit of RNA polymerase I, that led to rRNA synthesis inhibition and to MDM2 block. As shown for other cancer cell models, MDM2 inhibition by nucleolar stress downregulated E2F1 protein levels both in *p53-wt* and *p53-null* TGCT cells with the concomitant upregulation of unphosphorylated pRb. Finally, we show that sempervirine is able to enter the nucleus and accumulates within the nucleolus where it binds rRNA without causing DNA damage. Our results identify semperivirine as a novel rRNA synthesis inhibitor and indicate this drug as a non-genotoxic anticancer small molecule.

## Introduction

Sempervirine (2,3,4,13-tetrahydro-1*H*-benz[*g*]indolo[2,3-*a*]quinolizin-6-ium) is an alkaloid compound found as a constituent of *Gelsemium sempervirens* (Loganiaceae), a green-leaved plant that is under medicinal as poisonous plants. Previous reports explored the role of sempervirine as an anticancer drug, showing dramatic effects on cancer cell growth both in vitro and in vivo^1,2^. Recently, in a biomolecular screening, sempervirine has been identified as a selective inhibitor of murine double minute 2 (MDM2) ubiquitin ligase activity and it has been evidenced a role in inducing apoptosis in *p53-wt* cancer cell lines^3^.

MDM2 is a RING finger E3 ubiquitin ligase that negatively regulates p53 levels by promoting its proteasome-mediated degradation, thus inhibiting p53-mediated transactivation of target genes involved in DNA damage repair, cell cycle arrest, apoptosis, and senescence^4^. Among a myriad of upregulated genes, p53 induces the transcription of *MDM2* (or *HMD2*) itself, creating a negative feedback loop to limit the function of p53^5^. *MDM2* can be overexpressed in tumors with *p53-wt* and it is overamplified in several histological types, such as sarcomas, glioblastomas, bladder carcinomas, cholangiocarcinomas, and testicular germ cell tumors (TGCT)^6^. The C-terminal of MDM2 can bind the C-terminal of the highly related protein MDMX (HDMX or MDM4). Although MDMX does not have any E3 ligase activity, the MDM2–MDM4 heterodimer shows an optimal structure for E2-dependent p53 ubiquitination compared to MDM2 homodimers^7^.

Among solid neoplasias, TGCTs are the most frequent tumors that affect young men^8^. TGCT therapy is based on the stage and histology of the tumor and cisplatin, or platin derivatives are the first choice drugs, when chemotherapy is needed. Mortality from TGCT is due to tumor resistance to platin-based chemotherapy and the failure to clear all residual sites of disease after chemotherapy in the early treatment stages^8^. In vitro, cisplatin has been shown to be cytotoxic in human TGCT cell lines by inducing massive apoptosis^9–11^ and in response to platin chemotherapy a crucial role is played by p53, that following induction and posttranslational modifications activates the apoptotic pathway response^12^.

TGCT are sensitive to cisplatin chemotherapy, however, a fraction of treated patients develops cisplatin resistance. Even if cisplatin has been shown to induce p53 response, in these tumors its resistance is not directly linked to p53 status, since it is rarely mutated or deleted. The frequency of amplification of *MDM2* in TGCT, its mutually exclusive expression pattern with p53 mutations, and the ability to abrogate *wt* p53 function make MDM2 an attractive target for the development of novel antitumor agents. One of MDM2-specific inhibitor is nutlin-3a (thereafter called nutlin)^13^ that has been shown to cooperate with DNA damage to induce apoptosis in TGCT cells^14^. In agreement with this notion, nutlin treatment enhances cisplatin toxicity in lung cancer, ovarian cancer, and sarcoma cell lines^15,16^. However, its poor bioavailability, high toxicity, and its limited effects on MDMX-overexpressing cells^17^ have made it a poor candidate to the clinic translation.

In an attempt to identify new small molecules that could target p53/MDM2 axis in TGCTs with better bioavailability, we tested sempervirine in in vitro assays. Unexpectedly, we found that the drug not only targeted *p53*-*wt*, but also *p53-mutated* or *p53-null* tumor cell lines, while it was ineffective on non-transformed cells, and it significantly reduced cisplatin concentration in cytotoxic assays of resistant cells. Sempervirine not only increased p53 levels, as expected by its inhibitory activity on MDM2, but it also induced RPA194 (the catalytic subunit of RNA polymerase I (RNA Pol I)) degradation and nucleolar stress in *p53-wt*, *p53-mutated*, and *p53-null* TGCT cells. We found that RPA194 stability was dependent either on MDM2 levels or on its activity. Sempervirine preferentially bound to nucleolar rRNA without inducing DNA damage, supporting a role of sempervirine as ribosome biogenesis inhibiting agent. The ability of sempervirine to hit tumor, but not normal cells through RPA194 degradation, and its synergistic effect with cisplatin highlights its selectivity and versatility as an antitumoral agent.

## Results

### Sempervirine induces cell cycle arrest and cell death in *p53-wt* and *p53-null* germ cell tumor lines

**Fig. 1:**
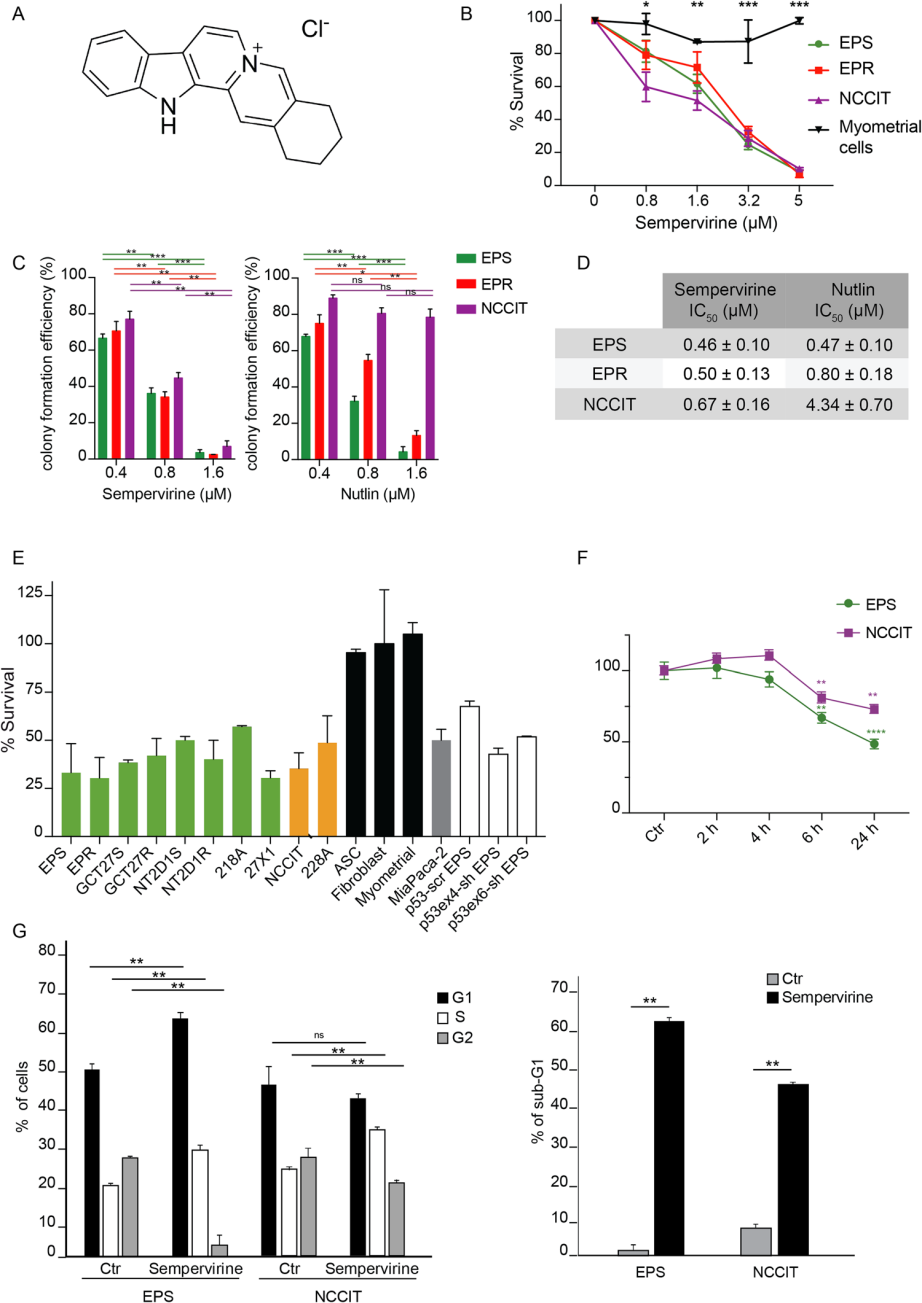
Sempervirine induces cell death in *p53-wt* and *p53-null* germ cell tumor lines. **A** Chemical structure of sempervirine chloride. **B** Mean half-maximal inhibition of cell survival/proliferation (IC_50_) of sempervirine after 72 h of culture in 2102EP(S) (EPS), 2102EP(R) (EPR), NCCIT, and myometral cells. *p < 0.05, **p < 0.01, ***p < 0.001, unpaired *t* test, number of biological replicates (*n*) *n* = 5. **C** Colony formation assay on 2102EP(S) (EPS), 2102EP(R) (EPR), and NCCIT after treatment with 0.4, 0.8, and 1.6 μM of sempervirine and nutlin, respectively. ****p < 0.0001, ***p = 0.0006. Two-way ANOVA, Tukey’s multiple comparisons test, *n* = 3. **D** IC_50_ of sempervirine and nutlin treatment in 2102EP(S) (EPS), 2102EP(R) (EPR), and NCCIT. **E** Proliferation assay on several TGCT cell lines *p53-wt* (green) and *p53-mut* or *-null* (yellow), MiaPaCa-2 (gray), control cell lines (black), and silenced 2102EP(S) (white) treated with 5 μM sempervirine for 24 h. Mean ± SD, *n* = 3. **F** Reversibility after 2, 4, 6, or 24 h of treatment with 5 μM sempervirine. **p < 0.01, ****p < 0.0001, paired *t* test, *n* = 3. **G** FACS analysis of sempervirine-treated 2102EP(S), and NCCIT cells showing the percentage of cells in G1, S, G2 cell cycle phases (left) and the percentage of sub-G1 cells (right) after 72 h of treatment. *p < 0.05, **p < 0.01, unpaired *t* test, *n* = 3.

Sempervirine (Fig. 1a) has been identified as a potent inhibitor of MDM2 E3 ligase activity on p53 in in vitro ubiquitylation assays, and as such it has been shown to activate the p53-mediated checkpoint and to inhibit cell growth in a p53-dependent manner. In the search of MDM2 E3 ligase inhibitors to block in vitro proliferation of TGCT cell lines, that are mostly *p53-wt*, we subjected 2102EP(S), 2102EP(R), and NCCIT, three embryonal carcinoma TGCT cell lines, to a proliferation assay in the presence of increasing doses of sempervirine. 2102EP(S) is a cisplatin-sensitive *p53-wt* TGCT line^18^, while 2102EP(R) has been derived from 2102EP(S) following long-term cisplatin treatment, to induce cisplatin resistance^19^. Despite sempervirine was initially identified as effective only in *p53-wt* cell lines, we also included NCCIT cells, a naturally occurring *p53-null* cell line, to test drug specificity. As control non-tumor cells, we used myometrial cells, adipose stromal cells (ASCs) and fibroblasts at early passages (p2–3). As shown in Fig. 1b and Supplementary Fig. 1c, cell survival similarly decreased in a dose-dependent manner 72 h after sempervirine treatment for each tumor cell type, irrespective of cisplatin sensitivity or p53 status. On the contrary, we did not find any significant antiproliferative effect on myometrial cells nor fibroblasts. In parallel experiments, we also evaluated the effects of nutlin, an inhibitor of p53–MDM2 interaction, on TGCT cell survival. As shown in Supplementary Fig. 1a, we found 2102EP(S) more sensitive compared with the parental cisplatin-resistant cells, while NCCIT and myometrial cells were insensitive (Supplementary Fig. 1a, b). We also performed the colony formation assay of TGCT lines in the presence of sempervirine or nutlin to test the mean half-maximal inhibition of cell survival/proliferation (IC_50_). As shown in Fig. 1c, colony formation was strongly inhibited by 1.6 μM sempervirine in all three cell lines irrespective of *p53* status, while nutlin, as expected, was effective only in *p53-wt* cells. Furthermore, the IC_50_ of sempervirine was comparable between the cell lines, ranging from 0.46 to 0.67 μM, while the IC50 of nutlin was 1.7-fold and 10-fold higher in 2102EP(R) and in NCCIT, respectively, compared to 2102EP(S) (Fig. 1d).

To further confirm the sensitivity of TGCT cells to sempervirine, we expanded the proliferation assay to other *p53-wt* cell lines (GCT27(S) and (R), NT2D1(S), and (R), 218 A, 27  X1), one additional *p53-mutated* cell line (228 A^20^, and a *p53-silenced* 2102EP(S) cell line (*sh-p53* EP(S)). As additional control for *p53*-mediated sensitivity, we included a pancreatic adenocarcinoma cell line (MiaPaCa-2) that carries the R248W mutation in p53. We observed that all the TGCT and pancreatic cell lines showed similar sensitivity to sempervirine at 5 μM concentration, regardless of the *p53* status and tumor type (Fig. 1e).

We then tested reversibility of sempervirine treatment by assessing cell survival after drug withdrawal at different time points. As shown in Fig. 1f, withdrawal of sempervirine 4 h after its addition did not induce significant cell death effects within the next 24 h of culture both in *p53-wt* and *-null* cells, while extending to 6 h of treatment >30% of 2102EP(S) and 20% NCCIT cells underwent cell death.

FACS analysis showed that the percentage of cells in G1 phase was increased at the expenses of G2 phase in sempervirine-treated 2102EP(S) cells after 72 h, while NCCIT were arrested in S phase, as expected for *p53* deficiency. Accordingly, the sub-G1 fraction of sempervirine-treated cells strongly increased for both cell types, although 2102EP(S) cells showed maximal response to sempervirine (Fig. 1g).

### Sempervirine and cisplatin synergize to suppress TGCT cell growth, and to induce p53 and MDM2 accumulation

**Fig. 2:**
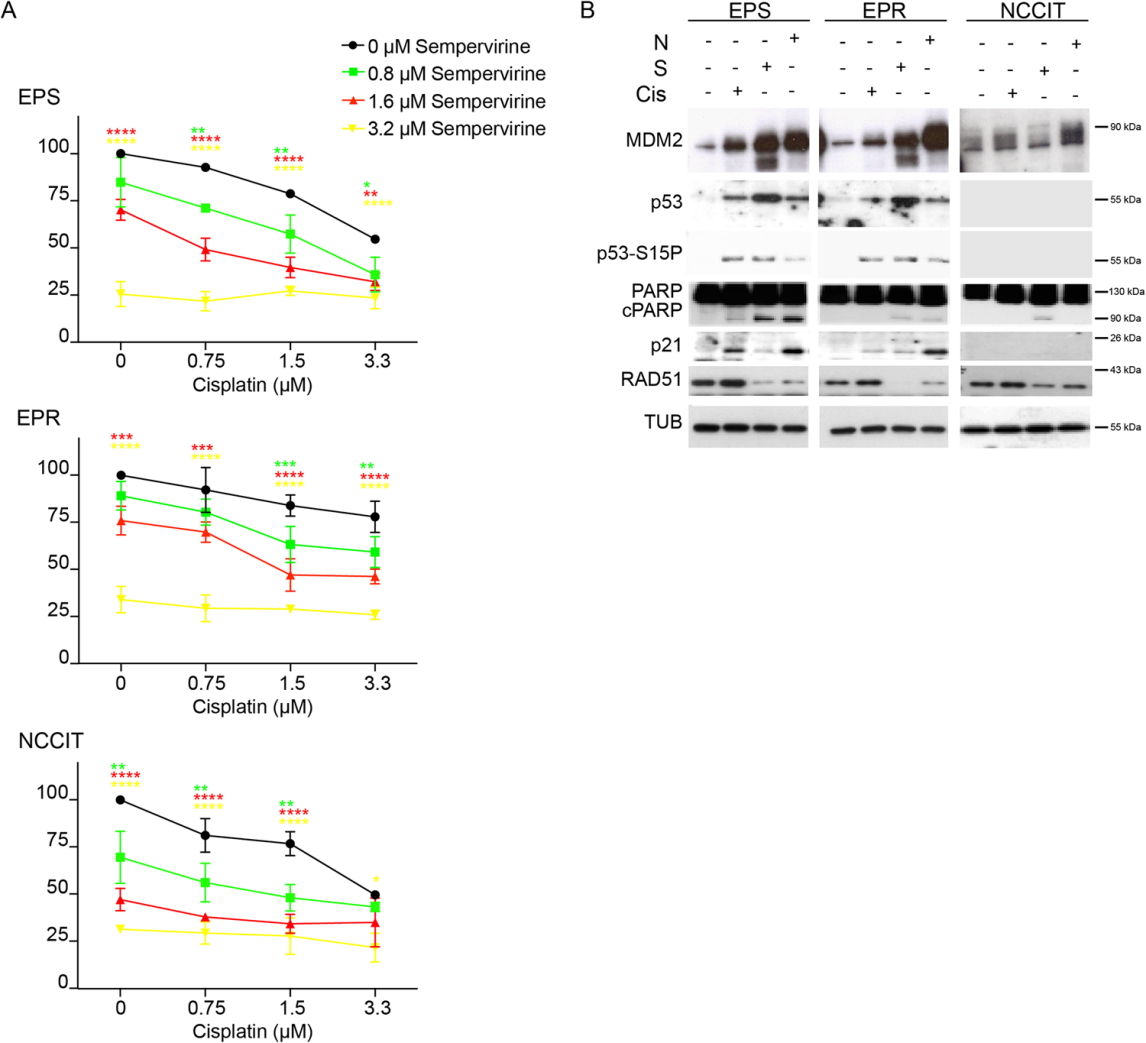
Combination of sempervirine and cisplatin affects TGCT cell growth and induces p53 and MDM2 accumulation. **A** 2102EP(S) (EPS), 2102EP(R) (EPR), and NCCIT sensitivity to different doses of cisplatin in combination with different doses of Sempervirine. *p < 0.05, **p < 0.01, ***p < 0.001, ****p < 0.0001, Two-way ANOVA, Dunnett’s multiple comparisons test, *n* = 3. **B** Western blot analysis of 2102EP(S) (EPS), 2102EPR (EPR), and NCCIT cell line extracts after 24 h of treatment with sempervirine, cisplatin, or nutlin, for MDM2, p53, and p53-S15P, PARP, cPARP, p21, and RAD51. Tubulin is the reference gene; *n* = 3.

To understand if treatment of TGCT cells with sempervirine might be functional to increase their response to cisplatin, we compared the survival of cell lines treated with cisplatin monotherapy and combined therapy with sempervirine. By a dose–response analysis, we calculated that the IC_50_ dose for cisplatin in 2102EP(S) and NCCIT cells was ~3.3 μM, while at this drug concentration >70% of 2102EP(R) were still viable (Fig. 2a). Next, we treated either cisplatin-sensitive or resistant cells with scalar doses of both drugs for 72 h. Cisplatin sensitivity threshold (3.3 μM) was restored in 2102EP(R) cells when sempervirine was administered at 1.6 μM concentration, while it was lowered to 1.5 μM in 2012EP(S) and NCC1T cells (Fig. 2a). Similar results were obtained using cisplatin in combination with 1.6 μM nutlin (Supplementary Fig. 1d, e), although not effective in the *p53-null* NCCIT cell line (Supplementary Fig. 1f). As anticipated by its inhibitory effect on MDM2 ubiquitin ligase activity, sempervirine strongly increased p53 and p53Ser15 phosphorylation levels, and this effect was more evident compared to nutlin or to the DNA-damaging effect of cisplatin in *p53-wt* TGCT cells (Fig. 2b). Parallel to the increase of p53 levels, also MDM2 levels were upregulated by sempervirine in *p53-wt* cells. As expected, MDM2 levels were basally low in *p53-null* cells, consistent with the transcriptional role of p53 on MDM2 expression (Fig. 2b).

While nutlin was active only on *p53-wt* cells, sempervirine induced an increase of apoptosis and cPARP levels in all the three TGCT lines (Fig. 2b), that was more evident in 2102EP(S) line. Similarly to nutlin, we found that sempervirine significantly upregulated *p53* target gene expression, as shown by the increased levels of p21, BAX, DRAM, PUMA, GADD45, and TIGAR, but not of RAD51, in cisplatin-sensitive cells and to a minor extent in cisplatin-resistant *p53-wt* cells, while none of the genes were upregulated in *p53-null* cells (Supplementary Fig. 2a). To understand if the two other members of p53 family, p63 and p73, might mediate the cytotxic effects of sempervirine also on *p53-null* cells, we monitored their protein levels either in both the cell lines; however, we did not find any significant effect (Supplementary Fig. 2b). Sempervirine cytotoxicity was not mediated by DNA damage, since we did not find any increase of γH2AX foci, as revealed by immunofluorescence analysis (Supplementary Fig. 2c). Overall these data indicate that sempervirine can induce cell death by activating p53-dependent and independent pathways.

### Sempervirine treatment induces nucleolar stress in *p53-wt* and *-null* cells

**Fig. 3:**
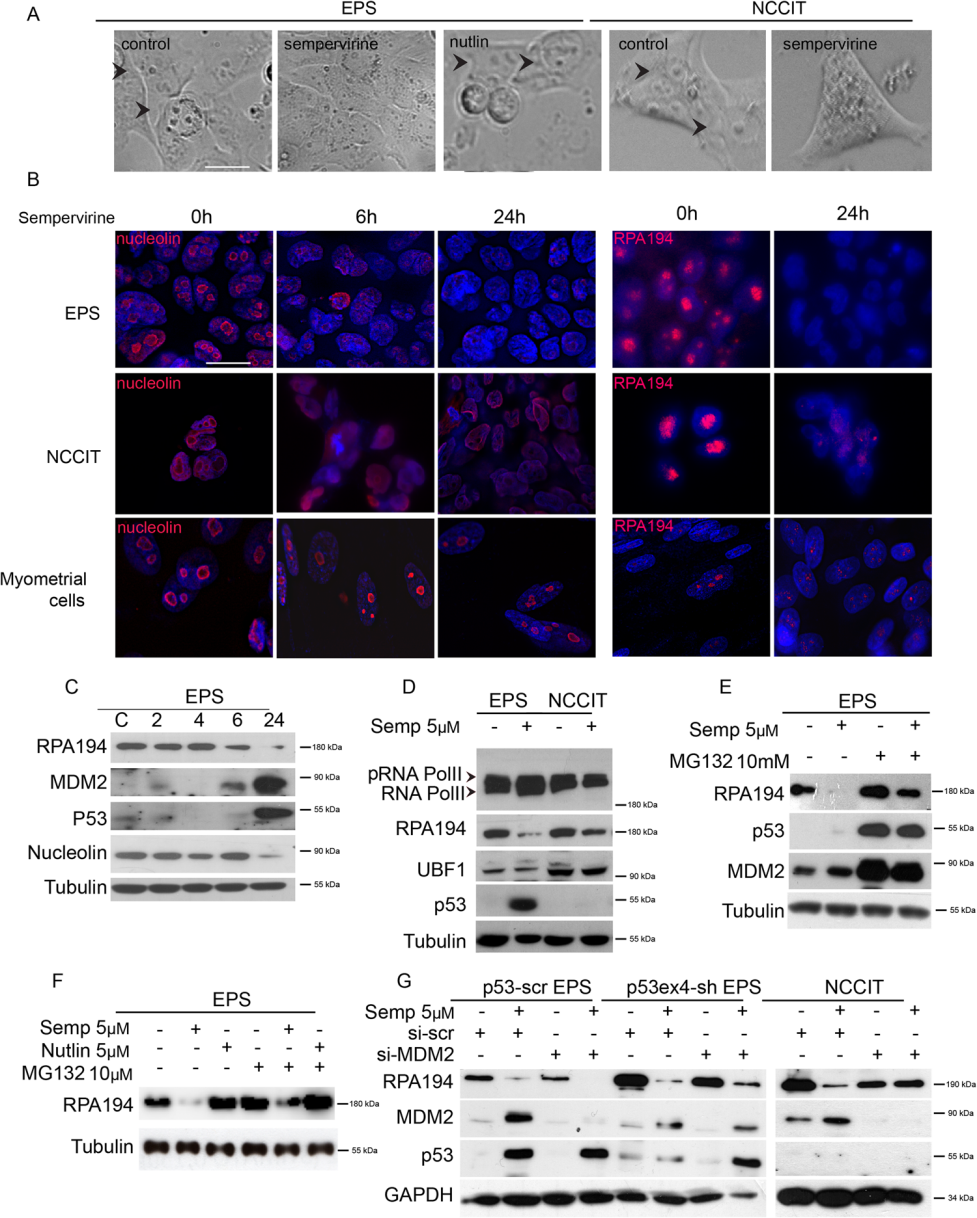
Sempervirine induces nucleolar stress in *p53-wt* and *p53-null* cells. **A** Light microscopy images of 2102EP(S) (EPS) and NCCIT nucleoli. Control, sempervine, or nutlin treatments were carried in EPS; control or sempervirine treatments were carried in NCCIT. Scale bar = 20 μM. Arrowheads pointing to nucleoli. **B** Immunofluorescence analysis on 2102EP(S) (EPS), NCCIT, and myometrial cells for nucleolin (left panel) at 0 h (left row), 6 h (middle row), 24 h (right row), and for RPA194 (right panel) at 0 h (left row) and 24 h (right row). Scale bar = 20 μM; *n* = 3. **C** Western blot analysis on extracts of 2102EP(S) (EPS) cell lines treated with 5 μM sempervirine at the indicated times, probed for RPA194, MDM2, p53, and nucleolin. Tubulin is the reference gene; *n* = 3. **D** Western blot analysis on cell extracts from 2102EP(S) (EPS) and NCCIT cell lines treated for 24 h with or without 5 μM sempervirine and probed for RNA Pol II, RPA194, UBF-1, and p53. Tubulin is the reference gene. Arrowhead points to the slower migrating band of RNA Pol II that corresponds to the phosphorylated form; *n* = 3. **E** Western blot analysis on cell extracts from 2102EP(S) (EPS) cell lines treated with or without 5 μM sempervirine in the absence or presence of 10 μM MG132. Extracts were probed for RPA194, p53, and MDM2. Tubulin is the reference gene; *n* = 3. **F** Western blot analysis on extracts from 2102EP(S) (EPS) cell lines treated for 24 h with or without 5 μM sempervirine or 5 μM nutlin in the presence or absence of 10 μM MG132 added for the last 6 h of culture, and probed for RPA194. Tubulin is the reference gene; *n* = 3. **G** Western blot analysis on cell extracts from sh-scrambled p53 2102EP(S) (*p53-scr* EPS) or sh-exon4 p53 2102EP(S) (*p53ex4-sh* EPS) cell lines that underwent MDM2 silencing with scrambled or MDM2 siRNAs (*si-scr* and *si-MDM2*, respectively) treated for 24 h with or without 5 μM sempervirine, and probed for RPA194, MDM2, and p53. GAPDH is the reference gene; *n* = 3.

**Fig. 4:**
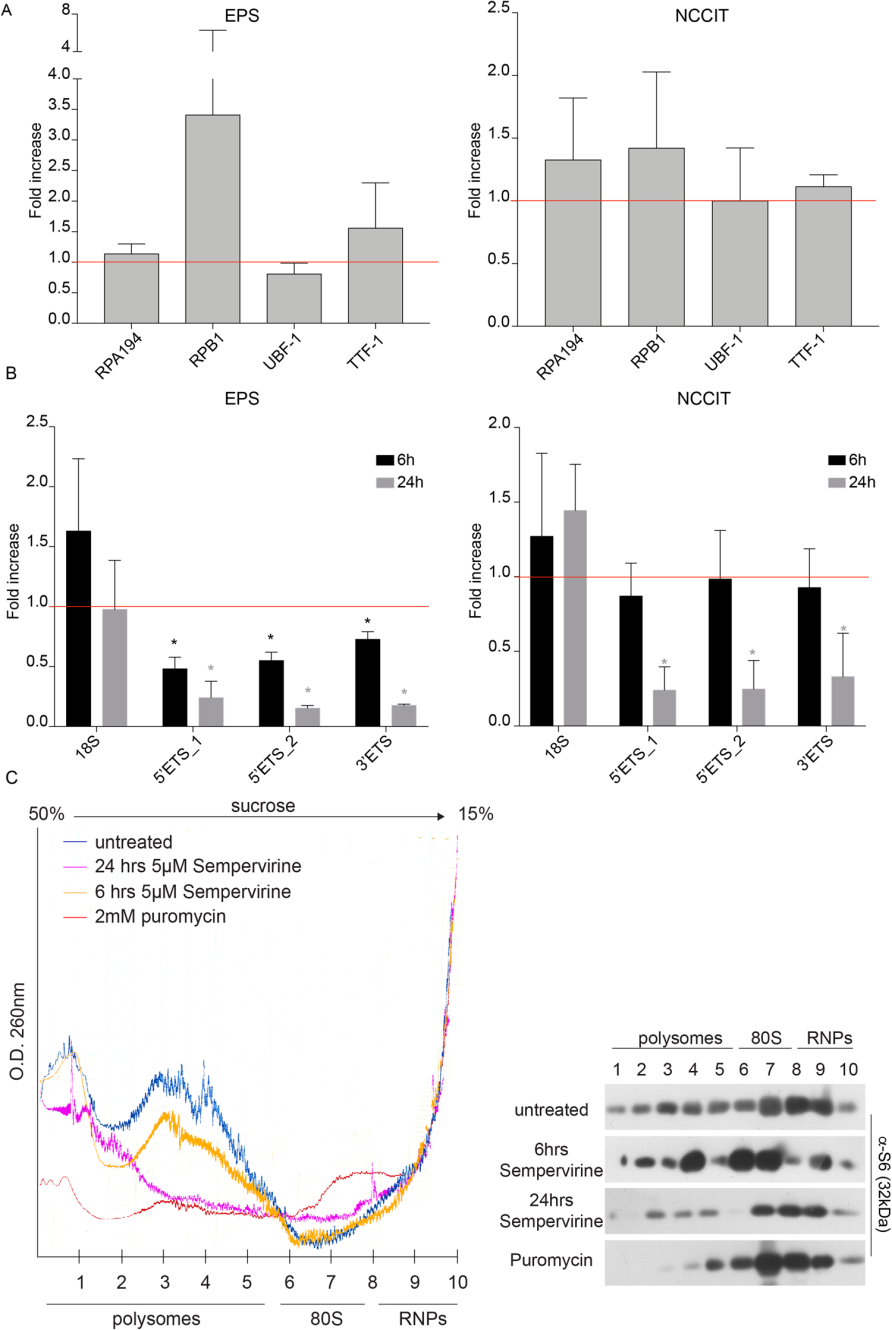
Sempervirine represses rRNA synthesis and ribosome assembly. **A** RT-qPCR for RPA194, RBP1, UBF-1, and TTF1 mRNAs from 2102EP(S) (EPS) or NCCIT treated for 24 h in the presence or absence of 5 μM sempervirine. Fold change is shown relative to the control (untreated 2102EP(S) (EPS) or NCCIT, represented by the red line, HPRT was used as reference gene). Mean ± SD, *n* = 3. **B** qRT-PCR for 18 S, 47S-specific 5′ETS (5′ETS_1), 18 S 5′junction (5′ETS_2), 18 S 3′junction (3′ETS) RNAs from 2102EP(S) (EPS), or NCCIT treated for 6 or 24 h with 5 μM sempervirine. Fold change is shown relative to the control (untreated 2102EP(S) (EPS) or NCCIT, represented by the red line, *HPRT* was used as reference gene). Mean ± SD, *n* = 3. *p < 0.05, paired *t* test. **C** Polysomes fractionation profiling of 2102EP(S) cells at 6 and 24 h after treatment with 5 μM sempervirine, or after 15 min with 2 mM puromycin (left panel) and western blot analysis of the relative fractions probed for S6 (right panel); *n* = 5.

Tumor cell lines are characterized by the presence of large and prominent nucleoli. By light microscopy, we noticed that the nucleolar compartments of either *p53-wt* or *p53-null* cell lines were almost completely disassembled after 24 h of sempervirine, but not after nutlin treatment (Fig. 3a). By immunofluorescence analysis, we found that nucleolin labeling redistributed from the granular component and dense fibrillar center of the nucleoli to the nucleus following 6 h of sempervirine treatment both in 2102EP(S) and NCCIT cells, and its levels were strongly decreased after 24 h of sempervirine treatment, as confirmed by western blot analysis (Fig. 3b, c). Also pancreatic cancer MiaPaCa-2 cells, that carry a mutated *p53* allele, showed a similar pattern of nucleolin staining, indicating that sempervirine-induced nucleolar stress independently from their *p53* status (Supplementary Fig. 3). As a control, we included human myometrial cells, that are insensitive to sempervirine (Fig. 1b) and did not show redistribution nor decrease of nucleolin levels (Fig. 3b). To understand if nucleolar stress could be a common mechanism for sempervirine to induce p53-dependent or independent cell death, we assessed if the drug might affect other nucleolar protein components. Indeed, we found that RPA194, the catalytic subunit of RNA Pol I, but not upstream binding-factor-1 (UBF-1) protein levels (Fig. 3c, d), were strongly downregulated following 24 h of treatment either in *p53-wt* or *-null* cells. To understand if sempervirine was affecting post-transcriptional regulation of RPA194, we also evaluated its mRNA levels and found that either RPA194 or UBF or TTF1, a factor involved in termination of rRNA transcription, were not affected (Fig. 4a). In support to these results, we found that total and phosphorylated protein levels for the catalytic subunit of RNA Pol II (RPB1), as well as its mRNA, were not affected, indicating that sempervirine does not inhibit mRNA transcription (Figs. 3d and 4a).

We found that the reduction of RPA194 was appreciable starting from 6 h of sempervirine treatment both in *p53-wt* and *p53-null* cells, and corresponded to the time point in which such treatment became irreversible (Figs. 1f and 3c). The decrease of RPA194 levels after 6 or 24 h of sempervirine treatment was partially reversed by the concomitant treatment with proteasome inhibitor MG132 added in the last 6 h of culture (Fig. 3e, f), while nutlin was not effective (Fig. 3f). Moreover, nutlin did not modify RPA194 levels (Fig. 3f), further confirming that the two drugs blocked MDM2 through different mechanisms of action. These results suggest that sempervirine activated rapid proteasome-mediated degradation of RPA194, inducing ribosomal stress.

To assess if MDM2 expression was also required to protect RPA194 stability, we transiently silenced MDM2 in either *p53-wt*, *p53* stably silenced cells (*p53-scr* EP(S), or *p53ex4-sh* EP(S)) or NCCIT cells (*p53-null*), with scr or MDM2 siRNAs. We found that RPA194 levels were reduced by MDM2 silencing in all the cell lines irrespective of p53 status, and sempervirine addition further reduced RPA194 levels (Fig. 3g). These results suggest that MDM2 protects RPA194 stability.

### Sempervirine represses rRNA synthesis

We then monitored if sempervirine was affecting rRNA synthesis rate as reflected by the levels of short-lived 5′ and 3′ external transcribed spacer (5′ETS and 3′ETS, respectively) rRNAs of the 47 S pre-rRNA. Primer sets were designed in the 18 S processed regions within the 47 S precursor RNA that undergo more rapid processing during rRNA synthesis. When testing *p53-wt* cells, we found a reduction of the 5′ETS and 3′ETS levels after 6 h of sempervirine treatment that was further decreased after 24 h, while the levels of 18 S subunit did not change. In *p53-null* cells, we found that 5′ and 3′ETS levels decreased only after 24 h of sempervirine treatment but not after 6 h, suggesting that RPA194 activity was more stable in *p53-null* cells (Fig. 4b). Altogether these results suggest that sempervirine induces instability of the 47 S precursor levels that is in line with the reduction of RPA194 protein.

Although 18 S RNA levels were not affected, we investigated if the translational apparatus might be influenced by sempervirine treatment. To this end, we performed ribosome profiling experiments in 2102EP(S) cells treated for 6 or 24 h with the drug. As shown in Fig. 4c, a slight decrease of the lighter polysome fractions was observed after 6 h of sempervirine, while the distribution of heavier fractions was almost comparable. An altered ribosomal profile was found after 24 h of sempervirine treatment, and the abundance of ribosomes engaged in polysomes was strongly reduced compared to control profiles (Fig. 4c). Moreover, we did not find any increase of the monosome fraction, while puromycin treatment, through premature chain termination, disassembled heavy polysomes fractions but not monosomes (Fig. 4c), suggesting that sempervirine induced ribosomal disaggregation. Similar results were obtained also in *p53-null* cells (NCCIT and *p53ex4-sh* EP(S), data not shown).

### Sempervirine binds rRNA

**Fig. 5:**
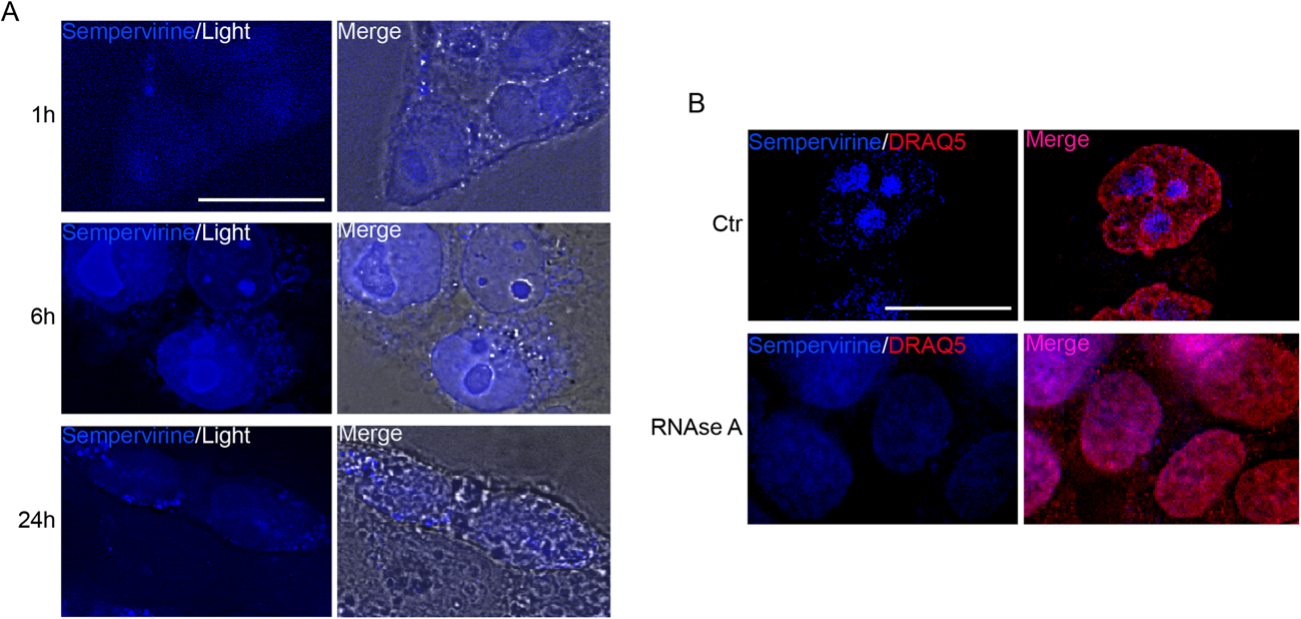
Sempervirine is a fluorescent molecule and preferentially binds to rRNA. **A** Fluorescence and light microscopy images of in vivo labeled NCCIT cells after 1, 6, and 24 h of incubation with 5 μM sempervirine. Scale bar = 20 μM. *n* = 3, 60 inspected fields. **B** Fluorescence images of fixed/permeabilized control NCCIT cells treated with or without 100 μg/ml RNAseA (15 min), and stained with sempervirine and Draq5 as nuclear counterstain. Scale bar = 20 μM. *n* = 2, 40 inspected fields.

BMH-21 and amodiaquine, two molecules that affect RNA Pol I stability, have been shown to intercalate DNA and affect RNA Pol I binding to rDNA^21,22^. Sempervirine is a fluorescent compound that binds both DNA and RNA with lower affinity compared to ethidium bromide, and shows a maximum emission at 440 nm wavenlength when excited in the ultraviolet spectrum^23^. Thus, using NCCIT as model cells, that show larger nucleoli compared to 2102EP cells, we asked whether sempervirine stains nuclei and nucleoli evenly. To this end, we treated NCCIT cells with 5 μM of the drug and then observed if nuclear labeling was occurring in vivo by fluorescence microscopy. Unexpectedly, we found that while cell nucleoplasm was faintly positive, nucleoli were brightly stained, suggesting that sempervirine was preferably binding to rRNA. The intensity of nucleolar labeling increased over time, being barely appreciable after 1 h and peaking at 6 h of incubation, the time point of irreversibility of sempervirine cytotoxic effect, while following 24 h of culture nucleolar fluorescence disappeared due to nucleolar disassembly (Fig. 5a). To confirm that sempervirine was predominantly bound to nucleolar RNA, we treated fixed-permeabilized NCCIT cells with 100μg/ml RNAse A for 15 min, to degrade single-strand RNA. DNA was counterstained using Draq5, a dye that specifically stains double-stranded DNA. As shown in Fig. 5b, while control fixed cells showed brilliant nucleoli labeled by sempervirine, nucleolar staining was completely abolished in RNAse-treated cells, confirming that sempervirine associated to rRNA within nucleoli.

### E2F1/pRB pathway is involved in sempervirine-induced nucleolar stress

**Fig. 6:**
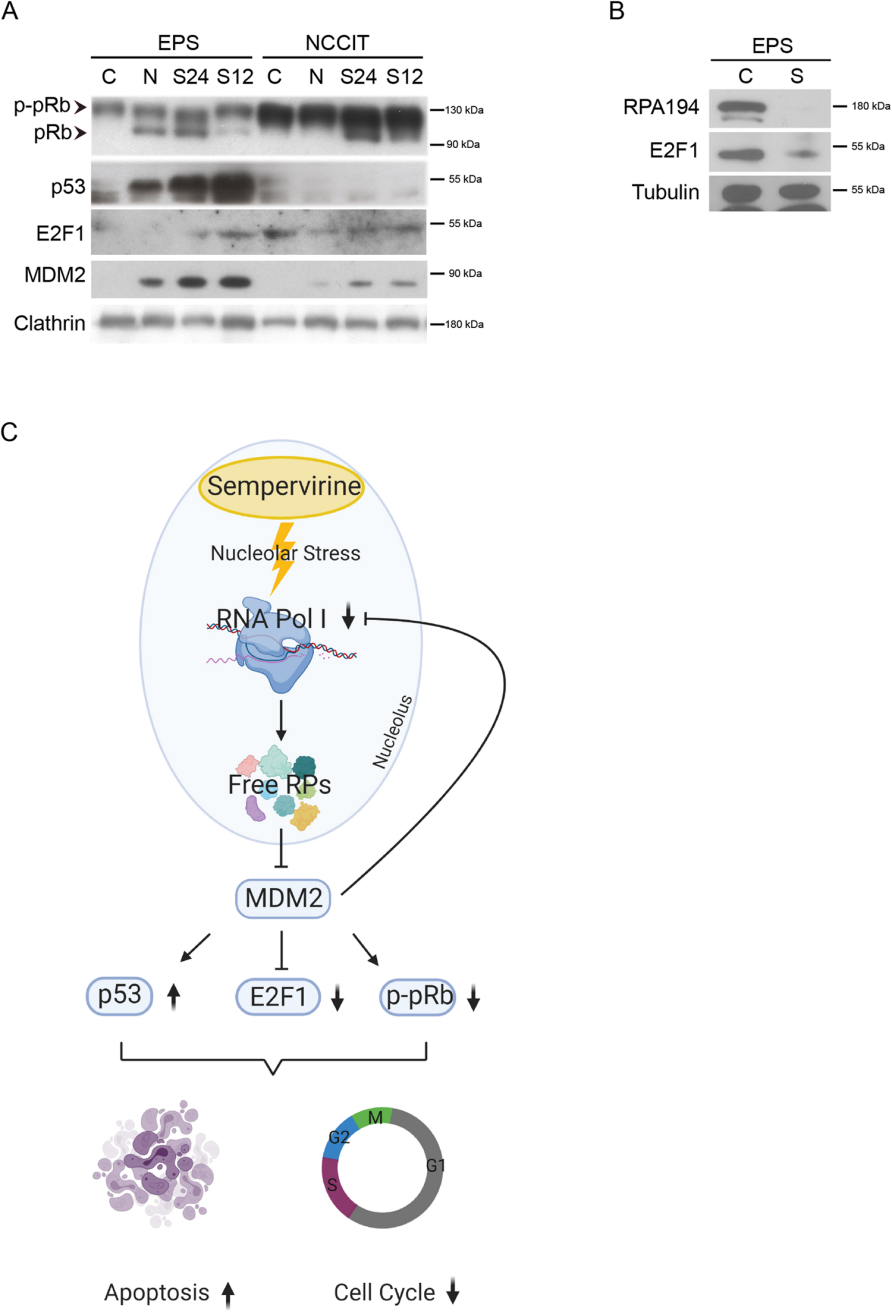
MDM2 inhibition causes E2F1 protein downregulation and pRb increase in *p53-wt* and *p53-null* cells. **A** Western blot analysis on cell extracts from 2102EP(S) (EPS) and NCCIT cell lines treated for 12 and 24 h with or without 5 μM sempervirine or 5 μM nutlin probed for pRB, E2F1, p53, and MDM2. Clathrin is the reference gene. Arrowheads point to the two bands that correspond to a faster migrating band (unphosphorylated form, pRB) and a slower migrating band (phosphorylated form, p-pRB) of p130 retinoblastoma; *n* = 3. **B** Western blot analysis on cell extracts from 2102EP(S) (EPS) cells treated for 24 h with or without 5 μM sempervirine, and probed for E2F1. Tubulin is the reference gene; *n* = 3. **C** Schematic representation of sempervirine mechanisms of action: sempervirine binds to nucleolar RNA and affects RPA194 stability, leading to nucleolar stress and MDM2 inhibition. Arrows indicate the concomitant activation of cell death and cell cycle arrest pathways.

Nucleolar stress cascade leads to the release of ribosomal proteins that sequester and inhibit MDM2, thereby increasing p53 stability^24^. Since we found that also *p53-null* cells underwent cell cycle arrest and nucleolar stress, we investigated which mechanism/s behind sempervirine might be acting in this setting. We reasoned that a potential candidate might be the E2F1/pRB pathway, that links ribosome biogenesis to cell cycle^25,26^. We found that E2F1 levels were decreased following sempervirine treatment both in *p53-wt* and *p53-null* cells, while the unphosphorylated form of pRb increased in both cell types (Fig. 6a, b). Although a similar effect on E2F1 with nutlin treatment was observed also in *p53-null* cells, in these latter, we did not find any increase of unphosphorylated pRb levels (Fig. 6a).

## Discussion

To reinforce tumor-suppressor functions of p53, much effort has been devoted in the recent years to the development of molecules that uncouple it from MDM2 control. Among these, nutlins have been developed that prevent MDM2 binding to p53 in the p53-binding pocket, leading to p53 accumulation, cell cycle arrest, and apoptosis in *p53-wt* cancer cells^13^. The hematologic toxicity and the poor bioavailability of nutlins have made this class of drugs of difficult employment in the treatment of cancer, and new molecules are being developed to overcome this issue. By modulating the E3 ubiquitin ligase activity of MDM2, other small molecules have been isolated that block MDM2 autoubiquitylation, leading to increased p53 stability^27^. Sempervirine has been previously identified as an MDM2 E3 ligase inhibitor that stabilizes p53 and induces cell death in U2OS cells^3^; however, reports on its efficacy in killing cancer cells are scant and not well described^1^. We first investigated if sempervirine could inhibit cancer cell proliferation by targeting MDM2/p53 axis, paralleling its effects with those of nutlin on TGCT cell lines. While the drug inhibited cell growth in a wide panel of *p53-wt* TGCT cells, we found that, differently from nutlin, sempervirine also arrested *p53-null* TGCT and *p53-mutated* cancer cells. Interestingly, we found that the drug sensitized both *p53-wt* and *p53-null* cells to cisplatin treatment, restoring to 3.3 μM the concentration needed to reach cisplatin IC_50_ in resistant cells, and reducing it to 1.5 μM in *p53-wt* and *p53-null*-sensitive cells. We found that similarly to nutlin, sempervirine induced a consistent increase of p53 and MDM2 levels and of p53-inducible cell death genes in *p53-wt* cells. MDM2 is not only a transcriptional target of p53, but its protein levels can be also regulated by autoubiquitylation. Since we observed an increase of MDM2 also in *p53-null* cells, this suggested that the inhibition of MDM2-ubiquitylating activity by sempervirine triggered p53-independent mechanism/s of cell death. Apart from p53 degradation, that is the best-characterized function, MDM2 plays also a role in the control of p63 and p73 stability^28–30^, as well as of cell cycle-related proteins, such as myc, p21^31^, or E2F1^32^. In the search for a common mechanism of action, we evaluated if these players might be modulated by sempervirine treatment. We did not find any effect on the expression levels of the other members of p53 superfamily nor of myc (data not shown) and we excluded that, at the concentration used in our experiments, sempervirine induced DNA damage as revealed by the lack of γH2AX histone foci. The lack of p21 induction in *p53-null* cells by sempervirine treatment also ruled out the possibility that MDM2 inhibition enhanced p21 stabilization leading to cell cycle arrest, at least for *p53-null* cells.

Several studies have shown that MDM2 protects E2F1 from SCF^SKP2^ ubiquitylation and degradation through p53-independent mechanisms^33,34^. Moreover, MDM2 silencing or inhibition by ribosomal stress both downregulate E2F1 levels in *p53-null* cells, leading to cell cycle arrest^34^. Ribosome-associated proteins (RP) RPL5, RPL11, and RPL23, released during ribosomal stress, all bind the central domain of MDM2 and apart from inhibiting p53 ubiquitylation, they prevent MDM2 ability to protect E2F1 degradation inducing cell cycle arrest and cell death^24,35–38^. Thus, RP release during ribosomal stress can activate cell cycle arrest via p53-dependent or p53-independent mechanisms^26^. RNA Pol I silencing has been shown to induce ribosomal stress that leads to cell cycle arrest through downregulation of E2F1 in the presence of increased levels of unphosphorylated pRb. Since we found that sempervirine treatment downregulated E2F1 levels both in *p53-wt* and *p53-null* cells with the concomitant upregulation of the unphosphorylated form of pRb, we hypothesized that apoptosis and cell cycle arrest induced by the drug might be linked to ribosomal stress. It is interesting to note that pRb has been shown to inhibit Pol I transcription by interacting with UBF^39^, and/or binding to the rDNA promoter^40^. Indeed, sempervirine induced nucleolar disruption and, most importantly, it promoted the degradation of RPA194, but not of UBF, nor of RNA Pol II catalytic subunit, suggesting that sempervirine interfered with rRNA but not mRNA transcription. The decrease of RPA194 protein levels was evident after 6 h of sempervirine treatment, a time point at which, when removed from culture, its cytotoxic effects were considered irreversible, even if proteasome inhibition could partially rescue RPA194 degradation. Sempervirine has been referenced as an MDM2 inhibitor on the basis of chemioluminescent methods that only reported the amount of autoubiquitylation and p53 ubiquitylation as a readout of MDM2 inhibition^3^. In our experiments, we observed that sempervirine affected RNA Pol I stability by increasing RPA194 sensitivity to proteasome degradation. This event led to the reduction of precursor-specific regions of 47 S rRNA that occurred more rapidly in *p53-wt* compared to *p53-null* cells, indicating that sempervirine was affecting ribosome biogenesis. As a result, polysome profiling showed a slight decrease of the heavy complexes following 6 h of sempervirine that completely disappeared after 24 h of treatment strongly supporting ribosomal stress, as the cause of MDM2 inhibition and cell death via p53-dependent and independent pathways. To date, few drugs have been demonstrated to induce RPA194 degradation, in particular BMH-21 and the antimalaric drug amodiaquine^21,22,41^ that cause RNA Pol I stalling by intercalating with rDNA, without causing DNA damage effects. Hernandonine, another natural small molecule with anticancer activity, has also been shown to destabilize RPA194 in a proteasome-dependent manner and to inhibit nascent rRNA synthesis, however, it shows DNA damage effects^42^. Although sempervirine has been shown to bind DNA and RNA in vitro, we found that in vivo it preferentially bound nucleolar RNA, as revealed by in vivo labeling experiments and by RNAse A digestion. The increase of fluorescence intensity during incubation suggested that sempervirine does not freely diffuse through the cell membrane and it reaches the maximal concentration within the cell at the same time point, when the drug cytotoxicity becomes irreversible. To our knowledge, this is the first report of a molecule that is able to induce RPA194 destabilization by interacting with rRNA without inducing DNA damage. We suggest that following accumulation within the nucleolus, sempervirine hinders RNA Pol I progression and causes rRNA transcription pausing, making RPA194 sensitive to proteasome degradation. MDM2 plays an important role in this scenario since its inhibition, by ribosomal stress or its depletion, by gene silencing, are both able to decrease stability of RPA194 pointing for a protective role of MDM2 against RPA194 proteasomal degradation. It has been previously shown that MDM2 block following rRNA synthesis inhibition is responsible for the reduction of E2F1 expression and for the increase of unphosphorylated pRB levels that arrest cycle progression^26^. Following sempervirine-mediated MDM2 block, we found that E2F1 and pRB levels are inversely regulated both in *p53-wt* and *p53-null* TGCTs, suggesting that E2F1/pRB pathway is activated independently from p53, but can reinforce p53 pathway in *wt* cells. In summary, we found that sempervirine, an anticancer small molecule previously described as an MDM2 inhibitor, actually is a potent inducer of nucleolar stress by destabilizing RPA194 in a proteasome-dependent manner, inhibiting nascent rRNA synthesis and disrupting ribosomal content (Fig. 6c). Our results support the use of sempervirine as a RNA Pol I inhibitor, and indicate this drug as a non-genotoxic anticancer small molecule. The evidence that sempervirine can restore sensitivity to cisplatin in resistant tumor cells without inducing DNA damage, indicates that RNA Pol I targeting can parallel other therapeutic approaches to kill cancer cells.

## Materials and methods

### Cell culture

Ten TGCT cell lines were used in this study. NT2D1, 218 A, 27X1, NCC1T, and 228 A were provided by R.S.K. Chaganti (Memorial Sloan-Kettering Cancer Center). GCT27 cisplatin-sensitive GCT27(S) and cisplatin-resistant GCT27(R) were provided by F. Viñals (Universitat de Barcelona, L’Hospitalet de Llobregat, Barcelona, Spain)^43^. 2102EP(S) and 2102EP(R) were provided by M. Höpfner (Universitätsmedizin Berlin, Germany). Cisplatin-resistant NT2D1 sublines were generated in our laboratory over a time period of 9 months by intermittent exposure to 1 µM cisplatin. After 6 h exposure, the cisplatin was removed from the culture medium and cells were allowed to recover 1 week until the next exposure. Myometrial cells have been described in^44^. Fibroblasts were a generous gift of Dr. R. Carrozzo (Dept. of Neuroscience, Bambino Gesù Children Hospital, Rome Italy). PT-5006 ASCs were from Lonza (Basel, Switzerland).

TGCT, MiaPaCa-2, myometrial, ASCs, and fibroblast cells were cultured in DMEM high glucose (11960044 Gibco, Thermo Fisher Scientific, Waltham, Massachusetts, US) supplemented with L-glutamine (25030081 Gibco, Thermo Fisher Scientific, Waltham, Massachusetts, US), Pen/Strep (10378016 Gibco), and 10% FBS (10270106 Gibco). Cells were grown in a 37 °C humidified atmosphere of 5% CO_2_.

### Chemicals and treatments

Sempervirine is a pentacyclic anhydronium indole alkaloid isolated from the roots of the North American shrub *G. sempervirens*, Alton, (Loganiaceae)^45,46^. The more stable salt form, i.e., sempervirine chloride, belongs to a natural products collection available at the Organic Chemistry Laboratory of the Department of Chemistry and Technology of Drugs (Sapienza University of Rome, Italy)^47,48^. The chemical identity of the molecule was assessed by re-running 1H NMR, and 13 C NMR experiments.

Sempervirine chloride: yellow solid, m.p.: 258–60 °C. ^1^H NMR (CD_3_OD, 400 MHz, 25°C, TMS): *δ* (ppm) = 8.820 (s, 1H, H-10); 8.528 (d, 1H, *J* = 7.2 Hz, H-11); 8.331 (d, 1H, *J* = 6.8 Hz, H-12); 8.284 (s, 1H, H-5); 8.139 (d, 1H, *J* = 8 Hz, H-4); 7.644 − 7.587 (m, 2H, H-1 and H-2); 7.387 − 7.348 (m, 1H, H-3); 3.160 (pseudo t, 2H, H-6, and H-6′); 3.008 (pseudo t, 2H, H-9, and H-9′); 1.991 (m, 4H, H-7, H-7′, H-8, and H-8′). ^13^C NMR (CD_3_OD, 100 MHz, 25 °C, TMS): *δ* (ppm) = 151.654, 142.655, 136.125, 135.272, 132.387, 131.145, 130.554, 127.346, 123.302, 122.716, 122.439, 122.169, 120.693, 117.165, 113.836, 30.726, 27.682, 23.066, 23.008. HRMS 273.14. ESI-MS (negative) *m*/*z*: 307.90 (calcd. for C_19_H_17_Cl 308.81).

Sempervirine was dissolved at a concentration of 10 mM in DMSO (A3672 Applichem, Darmstadt, Germany) and stored at −20 °C. Nutlin-3a (Selleck Chemicals, Verona, Italy) was dissolved at a concentration of 10 mM in DMSO and stored at −20°C. Cisplatin (Cis-diammineplatinum (II) dicloride P4394, Sigma-Aldrich) was dissolved in saline at 3.3 mM concentration and stored at −80 °C.

### p53 silencing and MDM2 siRNA transfection

Sh-scrambled (sh-scr), sh-p53ex4, and sh-p53ex6 sequences (see Table 1) were cloned into the pLKO.1 (#8453 Addgene, Watertown, Massachusetts, US) cloning vector using AgeI and EcoRI as restriction sites, and lentiviral packaging acquisition was performed into HEK293T cells. 2101EPS cells positively infected sh-scr, sh-p53exon4, and sh-p53exon6 were selected using (1 mg/ml) puromycin (P8833 Sigma-Aldrich; Supplementary Fig. 4).

**Table 1:** Cloning list.

Name	Sequence
AgeI-EcoRI sh-TP53ex4 for	5′-CCGGT-CC-GACTCCAGTGGTAATCTAC-TTCAAGAGA-GTAGATTACCACTGGAGTC-TTTTTT-G-3′
AgeI-EcoRI sh-TP53ex4 rev	5′-AATTC-AAAAAA-GACTCCAGTGGTAATCTAC-TCTCTTGAA-GTAGATTACCACTGGAGTC-GG-A-3′
AgeI-EcoRI sh-TP53ex6 for	5′-CCGGT-CC-ACTCCACACGCAAATTTCCTT-TTCAAGAGA-AAGGAAATTTGCGTGTGGAGT-TTTTTT-G-3′
AgeI-EcoRI sh-TP53ex6 rev	5′-AATTC-AAAAAA-ACTCCACACGCAAATTTCCTT-TCTCTTGAA-AAGGAAATTTGCGTGTGGAGT-GG-A-3′
AgeI-EcoRI sh-Scr for	5′-CCGGT-CC-TAAGGTTAAGTCGCCCTCG-CTCGAG-CGAGGGCGACTTAACCTTAGG-TTTTTT-G-3′
AgeI-EcoRI sh-Scr rev	5′-AATTC-AAAAAA-TAAGGTTAAGTCGCCCTCG-CTCGAG-CGAGGGCGACTTAACCTTAGG-GG-A-3′

RNAi experiments were performed using Lipofectamine 3000 Transfection Reagent (L3000015 Thermo Fisher Scientific) according to manufacturer’s instruction. For 2102EPS cells, we performed a double transfection using 1 mg of iMDM2 each time, for NCCIT, we performed a single transfection using 20 ng of iMDM2. iMDM2 and iCTR were purchased from Life Technologies (Thermo Fisher).

### Crystal violet assay

Cells were seeded in a 96-well plate and the next day incubated with the appropriate drug until the end of the experimental time. The medium was discarded, cells were washed with PBS (ECB4004L Euroclone, Pero, Italy), and dried under fume hood. A total of 100 μl of crystal violet solution (0.2% in H_2_O) was added in each well and after 30 min the plate was washed with water. The dry plate was used to measure the OD of each well using a plate reader (OD570 nm). The OD of the untreated cells is set to 100% and compared with the stimulated samples.

### Colony formation assay

Single-cell suspensions were plated in six well plates (100 cells/well 2102EPS, 2102EPR, NCCIT). After 1 day, cells were treated with sempervirine or nutlin at the indicated dose. After 12–14 days, the medium was discarded, and the plates were dried under a chemical hood. The colonies were stained with 2% methylene blue/0.1% crystal violet solution (M9140 and C0775, Sigma-Aldrich).

### Protein extraction and western blot analysis

Cells were extracted in 50 mM HEPES (H3375 Sigma-Aldrich), 1% Triton X-100 (T8787 Sigma-Aldrich), 100 mM NaCl (S7653 Sigma-Aldrich), 10 mM MgCl_2_(M8266 Sigma-Aldrich,) 10% glycerol (G5516 Sigma-Aldrich), 0.5 mM dithiothreitol (DTT) (D9779 Sigma-Aldrich), 10 mM β-glycerophosphate (G9422 Sigma-Aldrich), 0.1 mM sodium orthovanadate (450243 Sigma-Aldrich), and protease and phosphatase inhibitor cocktail (PPC1010 Sigma-Aldrich).

Antibodies used are listed in Table 2.

**Table 2:** Antibodies list and dilution.

Antibody name	Specification	Dilution
RPA194	sc-48385 (Santa Cruz Technologies)	1:1000–1:200
P53	sc-126 (Santa Cruz Technologies)	1:1000
MDM2	OP46 (Sigma-Aldrich)	1:500
UBF-1	sc-13125 (Santa Cruz Technologies)	1:1000
GAPDH	Ab9485 (Abcam)	1:2000
Tubulin	#T4026 (Sigma-Aldrich)	1:2000
Nucleolin	sc-8031 (Santa Cruz Technologies)	1:1000–1:200
Fibrillarin	sc-11336 (Santa Cruz Technologies)	1:1000
E2F1	sc-251 (Santa Cruz Technologies)	1:1000
BrdU	sc-32323 (Santa Cruz Technologies)	1:1000
PARP	sc-7150 (Santa Cruz Technologies)	1:1000
P63	Generous gift of Prof. A. Peschiaroli	1:1000
P73	Generous gift of Prof. A. Peschiaroli	1:1000
Rb	Sc-50 (Santa Cruz Technologies)	1:1000
RAD51	sc-9349 (Santa Cruz Technologies)	1:1000
RNA pol II	sc-899 (Santa Cruz Technologies)	1:1000
Clathrin	Ab-2731 (Abcam)	1:1000
γH2AX	#9718 (Cell Signaling Technologies)	1:500
S6	#2217 (Cell Signaling Technologies)	1:1000
Phospho-P53	#9284 (Cell Signaling Technologies)	1:1000
P21	sc-6246 (Santa Cruz Technologies)	1:1000

### Immunofluorescence

Cells were fixed in 4% PFA (157-8 Electron Microscopy Sciences, Hatfield, PA) solution in PBS, permeabilized with 0.1% Triton (X-100 Sigma-Aldrich) in PBS, and incubated with primary antibodies. Immunofluorescence for BrdU was performed fixing cells in 70% ethanol (51976 Sigma-Aldrich) in PBS and DNA was hydrolyzed with 1.5 M HCl (H1758 Sigma-Aldrich).

Antibodies used are listed in Table 2.

Sempervirine labeling in fixed cells was performed incubating cells with a solution 5 μM sempervirine in PBS. DAPI and DRAQ5 were purchased from Thermo Fisher (Monza, Italy).

RNase A (10109169001, Roche, Sigma-Aldrich) treatment was performed diluiting RNAse A in PBS at a concentration of 100μg/mg for 15 min.

### qRT-PCR

RNA was extracted from cells using the Eurogold TriFast reagent according to manufacturer’s instructions (EMR507100 Euroclone) and treated with RNase-free DNase (M0303 New England Biolabs, Ipswich, Massachusetts, US). A total of 2 μg of RNA was retrotranscribed with M-MLV reverse transcriptase and random primers (BIO-65050 Bioline, Cincinnati, Ohio, US). Five percent of the reaction was used as template or qRT-PCR analysis (SYBR Green method, BIO-92005 Bioline). Primers used are listed in Table 3.

**Table 3:** Primers list.

Name	Sequence 5′ → 3′
DRAM1 for	TGTCTGTGCTTCACTAATTTCCA
DRAM1 rev	TCACAGATCGCACTCACTACG
TIGAR for	CTCCAGTGATCTCATGAG
TIGAR rev	CATGGCCCTCAGCTCACTTA
P21 for	GGAGACTCTCAGGGTCGAAA
P21 rev	GGATTAGGGCTTCCTCTTGG
RAD51 for	ACTGCAACTCTCTGGGTTGT
RAD51 rev	GTTGTGGGCCAAAGCTTTCTT
GADD45 for	CCATGCAGGGAAGGAAAACTATG
GADD45 rev	CCCAAACTATGGCTGCACACT
BAX for	CCCGAGAGGTCTTTTTCCGAG
BAX rev	CCGCCCATGATGGTTCTGAT
RPA194 for	CTTCATTCTTCCACAGGGCA
RPA194 rev	CCGAAAGGAACACAACAGCA
UBF for	CACCCTGAGATGAGCAACCT
UBF rev	GCCGCACTTTGAGATACACC
TTF1 for	GCACAATCAAGCGGATGTACC
TTF1 rev	CTTGTCTGCACTCTCAATGC
HPRT for	TGCTGGATTACATCAAAGCACTG
HPRT rev	TCCACCAATTACTTTTATGTCCCCT
5’ETS_1 for	ATGGACGAGAATCACGAGCG
5’ETS_1 rev	CAGCCACGAACCCGACAC
5’ETS_2 for	GCCGCGCTCTACCTTACCTACC
5’ETS_2 rev	CAGACATGCATGGCTTAATCTT
3’ETS for	AGTCGTAACAAGGTTTCCGTAGG
3’ETS rev	CCTCCGGGCTCCGTTAAT
18 S for	CGCCGCTAGAGGTGAAATTCT
18 S rev	CGAACCTCCGACTTTCGTTCT
RPB1 for	TTGTAGTGAGGTTTGCGCCT
RPB1 rev	GTCTCTGGGTATTTGATGCCA

### Polysomes–RNPs fractionation by sucrose gradients

A total of 80% confluent cells were homogenized in lysis buffer (100 mM NaCl, 10 mM MgCl2, 30 mM Tris-HCl (T1503 Sigma-Aldrich) pH 7.4, 1 mM DTT, 30 U/ml RNasin (N2111 Promega) supplemented with 0.5% Triton X-100, protease inhibitor, and 0.5 mM orthovanadate. After 10 min of incubation on ice, the lysates were centrifuged for 10 min at 12,000 × *g* at 4 °C. A total of 2 mg of total proteins were loaded onto a 15–50% linear sucrose (0335 VWR) gradient and sedimented by centrifugation for 120 min at 37,000 r.p.m. in a Beckman SW41 rotor (Fullerton, CA). The optical density at 254 nm was measured continuously using UVcord photometer (LKB, Stockholm, SWE). Each gradient was collected in ten fractions, proteins were TCA precipitated from each fraction, and analyzed by western blot according to Barrios et al.^49^.

### FACS analysis

Eighty percent confluent cells were washed twice in PBS, collected, fixed with 70% ethanol, and stained with propidium iodide (P4864 Sigma-Aldrich). Cell cycle analysis was carried out using flow cytometry (FACSCalibur, BD Biosciences) according to manufacturer’s instructions.

### Statistical analysis

All analyses were performed with Prism 7 software. All the variables have been synthesized as mean and standard deviation or mean and standard error of mean, and tests were considered significant for relative values *p* < 0.05. All experiments were performed on at least three biological replicates.

## Supplementary information

Supplemental Figure 1

Supplemental Figure 2

Supplemental Figure 3

Supplemental Figure 4
