# Supplementary figures and images for: Sempervirine inhibits RNA polymerase I transcription independently from p53 in tumor cells
Source: Cell Death Discov. 2020 Oct 28;6:111. doi: 10.1038/s41420-020-00345-4 (PMC7595235; doi:10.1038/s41420-020-00345-4)

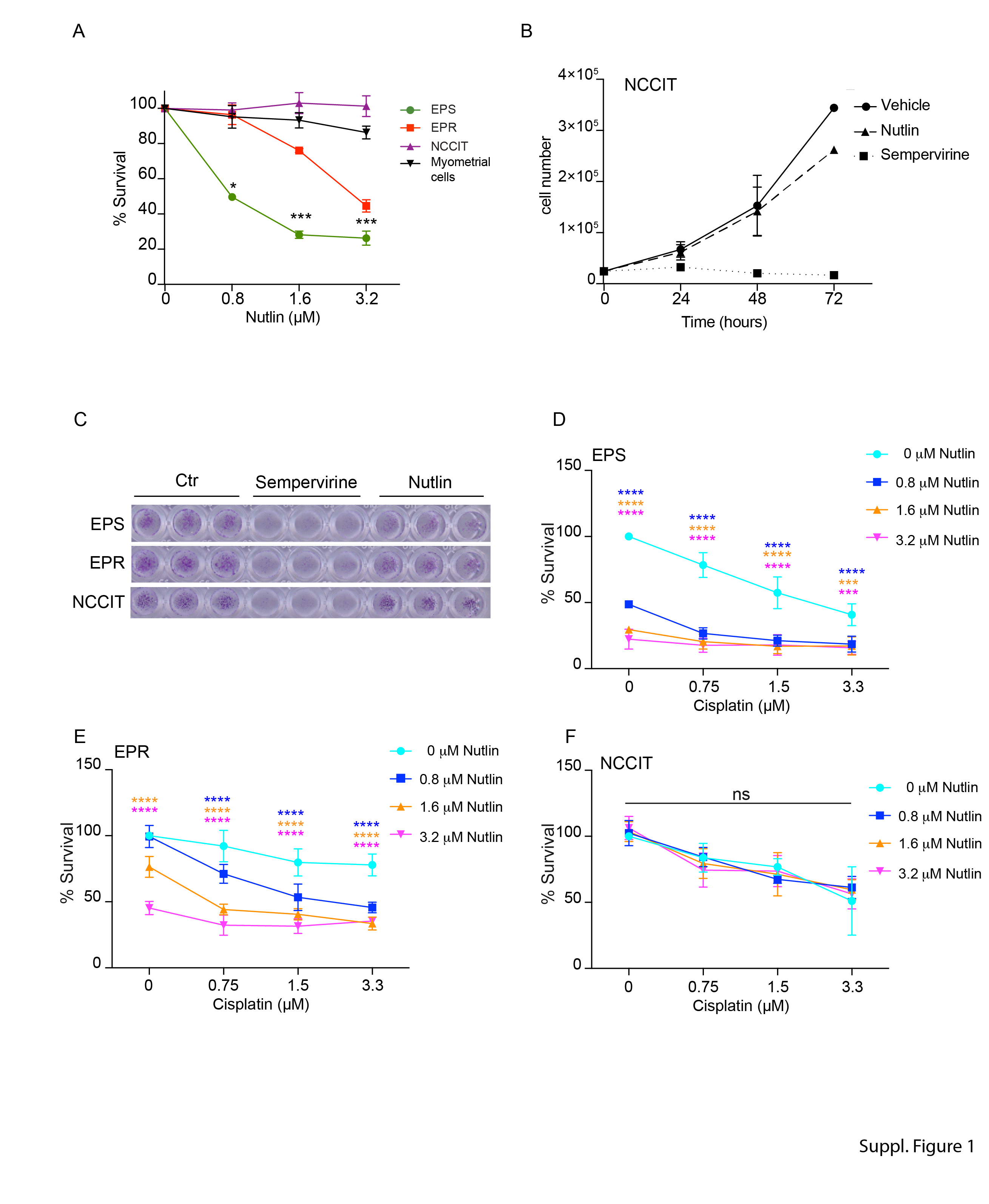

Supplement: Supplementary file 1 — Supplemental Figure 1 [file 41420_2020_345_MOESM1_ESM.tif]

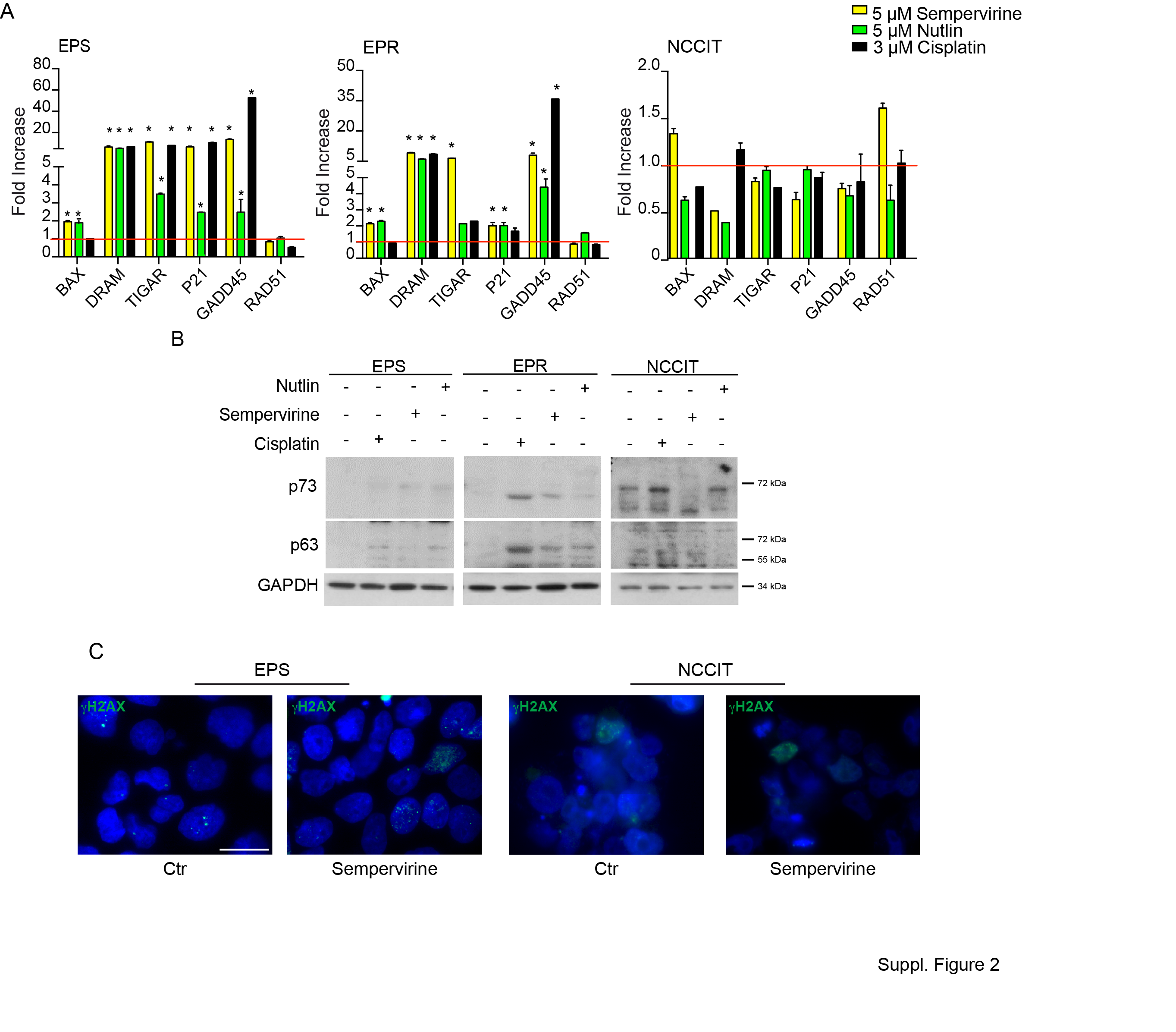

Supplement: Supplementary file 2 — Supplemental Figure 2 [file 41420_2020_345_MOESM2_ESM.tif]

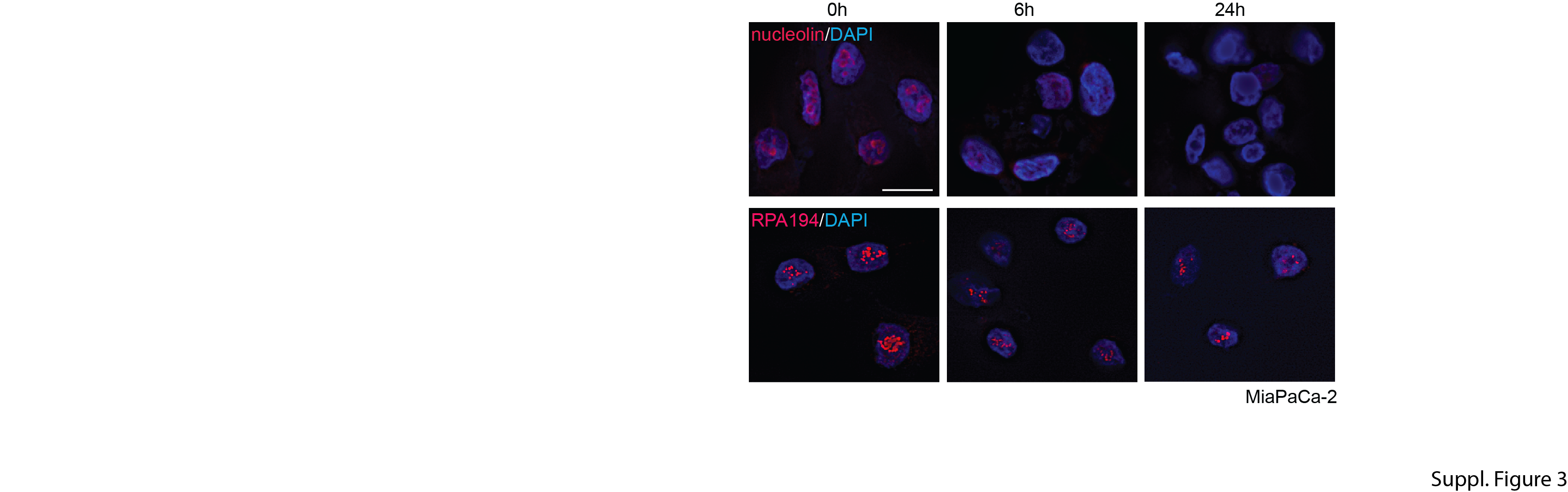

Supplement: Supplementary file 3 — Supplemental Figure 3 [file 41420_2020_345_MOESM3_ESM.tif]

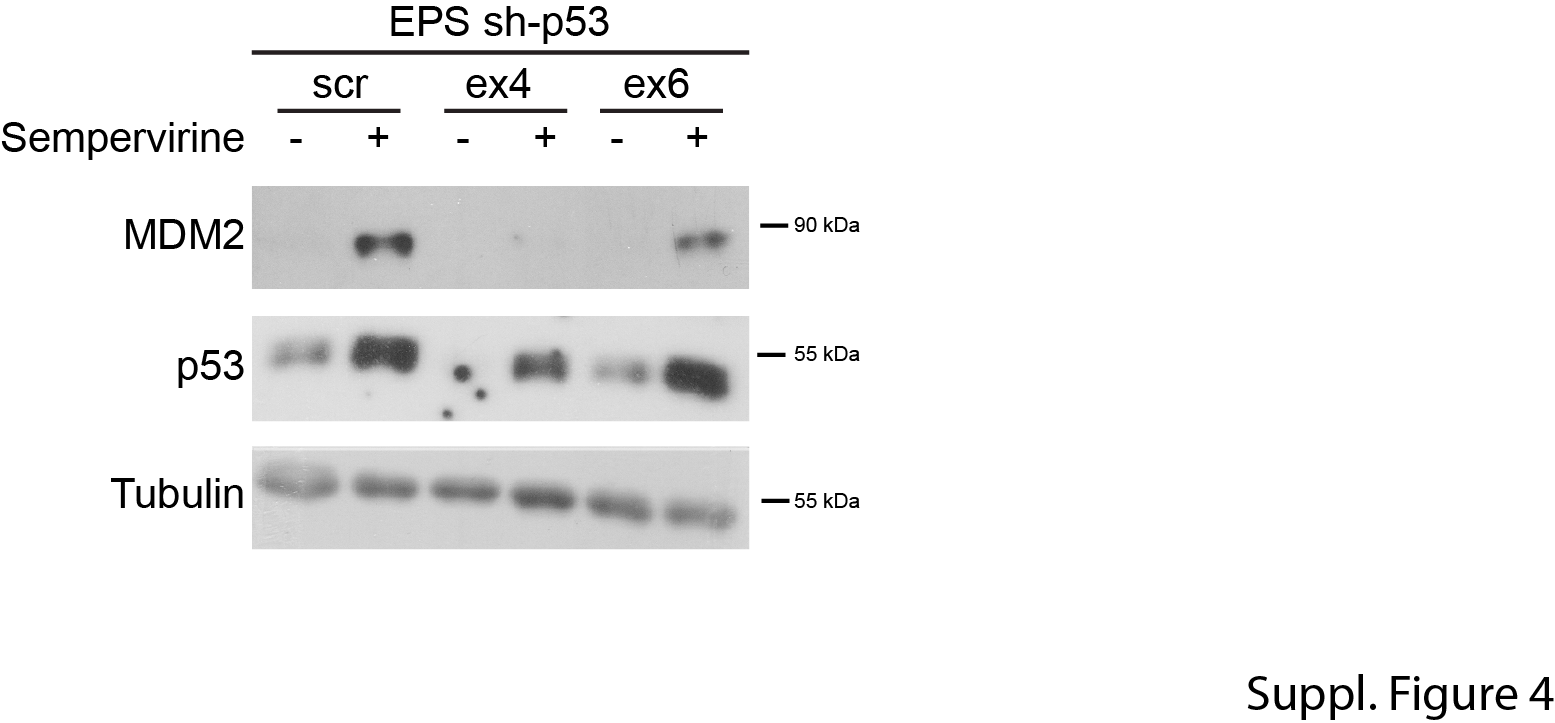

Supplement: Supplementary file 4 — Supplemental Figure 4 [file 41420_2020_345_MOESM4_ESM.tif]
